# Label-free hairpin-like aptamer and EIS-based practical, biostable sensor for acetamiprid detection

**DOI:** 10.1371/journal.pone.0244297

**Published:** 2020-12-23

**Authors:** Jianhui Zhen, Gang Liang, Ruichun Chen, Wenshen Jia

**Affiliations:** 1 Shijiazhuang Customs Technology Center P.R. China, Shijiazhuang, Hebei Province, China; 2 Beijing Research Center for Agricultural Standards and Testing, Beijing Academy of Agriculture and Forestry Science, Beijing, China; 3 Risk Assessment Lab for Agro-products (Beijing), Ministry of Agriculture, Beijing, China; 4 Beijing Municipal Key Laboratory of Agriculture Environment Monitoring, Beijing, PR China; Sichuan University, CHINA

## Abstract

Acetamiprid (ACE) is a kind of broad-spectrum pesticide that has potential health risk to human beings. Aptamers (Ap-DNA (1)) have a great potential as analytical tools for pesticide detection. In this work, a label-free electrochemical sensing assay for ACE determination is presented by electrochemical impedance spectroscopy (EIS). And the specific binding model between ACE and Ap-DNA (1) was further investigated for the first time. Circular dichroism (CD) spectroscopy and EIS demonstrated that the single strand AP-DNA (1) first formed a loosely secondary structure in Tris-HClO_4_ (20 mM, pH = 7.4), and then transformed into a more stable hairpin-like structure when incubated in binding buffer (B-buffer). The formed stem-loop bulge provides the specific capturing sites for ACE, forming ACE/AP-DNA (1) complex, and induced the *R*_CT_ (charge transfer resistance) increase between the solution-based redox probe [Fe(CN)_6_]^3−/4−^ and the electrode surface. The change of Δ*R*_CT_ (charge transfer resistance change, Δ*R*_CT_ = *R*_CT(after)_-*R*_CT(before)_) is positively related to the ACE level. As a result, the AP-DNA (1) biosensor showed a high sensitivity with the ACE concentration range spanning from 5 nM to 200 mM and a detection limit of 1 nM. The impedimetric AP-DNA (1) sensor also showed good selectivity to ACE over other selected pesticides and exhbited excellent performance in environmental water and orange juice samples analysis, with spiked recoveries in the range of 85.8% to 93.4% in lake water and 83.7% to 89.4% in orange juice. With good performance characteristics of practicality, sensitivity and selectivity, the AP-DNA (1) sensor holds a promising application for the on-site ACE detection.

## Introduction

In the past decades, pesticides have been widely used to control, kill or repel pests for improving the quality of agricultural products [1, 2]. However, the wide use of pesticides has led to inevitable consequences for Agro-products and environmental pollution [3]. Therefore, the pesticide residue detection is now a main concern of food safety experts, and a rapid, simple and accurate detection technique for pesticide is needed to keep humans from potential health risks [4].

Acetamiprid (ACE) is one of the new neonicotinoid class of systemic broad-spectrum and contact pesticide [5]. Until now, it has been widely employed as replacement insecticide of the conventional pesticide for controlling the insects on fruits, vegetables, and teas due to its relatively low chronic mammalian toxicity, and no long-term cumulative toxicity [6, 7]. But studies also show that ACE can generate potential health risks to human beings, who are exposed to the primary route of food, drinking water polluted by ACE [5]. The maximum residue limit for ACE in vegetables and fruits is set at less than 3 ppb (μg/g) by the U. S. Environmental Protection Agency (US EPA) [8]. In this regard, it is necessary to develop sensitive, selective, simple and reliable analytical tools to detect ACE in Agro-products, water and environment samples [9].

So far, different methods have been used for determination of ACE residues, mainly focused on the sophisticated chromatographic instruments, such as liquid chromatography-mass spectrometry (LC-MS) [10, 11], gas chromatography (GC) [12], liquid chromatography coupled with thermal lens spectrometric method [13], GC-MS/MS and UPLC-Q-Orbitrap systems [14], LC/ESI/MS [15], high performance liquid chromatography (HPLC), GC–MS/MS and UHPLC-MS/MS [16]. However, there are still some drawbacks, such as time-consuming for sample pre-treatment, high-price detecting equipments, and requiring highly qualified technicians and hazardous organic reagents [17]. Electrochemical DNA biosensor has been proposed as an efficient and promising alternative to conventional analytical methods due to its advantages, such as sensitivity, portability and stability [18–20]. Aptamers are short single stranded oligonucleotides which are generated by SELEX (systematic evolution of ligands by exponential enrichment) library that can bind specifically to target molecules [21]. And it has many outstanding advantages of inconsiderable toxicity and immunogenicity, inexpensiveness, feasible production, high thermal and chemical stability compared to antibody [22, 23]. And aptamer-based electrochemical sensors have been widely applied to detect toxic pollutants [24], such as pesticides [25], heavy metals [26], antibiotics [27, 28] and toxins [29, 30].

Recently, different types of biosensors for ACE detection have also been designed. For example, aptamer-based colorimetric sensing of ACE [31–33], label-free and enzyme-free fluorescent aptasensor for quantification of ACE [34, 35], aptamer induced AuNPs' catalytic effect for chemiluminescence (CL) detection of ACE [36], label-free electrochemical aptamer sensor for ACE residue determination [37–39]. The sensing principle of these sensors are based on the conformational change of AP-DNA after binding with ACE. These methods made great improvements in detection techniques for ACE. By comparison with other techniques, EIS-based DNA sensors possess many merits of separating the surface binding events from the solution impedance, less damage to the biological targets, and the possibility to use non-labeled DNA compared with the DPV/CV-based electrochemical biosensor [40]. EIS is very sensitive to the electrode DNA film, even for very small structural change or binding events of the modified DNA. However, to the best of our knowledge, little efforts have been given for investigating the AP-DNA conformation change before and after binding with ACE on the electrode surface.

In this work, an impedimetric label-free AP-DNA (1) sensor for the determination of ACE was reported. For the first time, we demonstrated that the Ap-DNA (1) modified on the gold electrodes transformed into a more stable secondary structure with stem-loop bulge (hairpin-like conformation) in binding buffer (B-buffer), forming a specific binding sites for ACE, thereby induced an increased charge transfer resistance change (Δ*R*_CT_). The sensing principle was further confirmed by CD and EIS. Therefore, ACE was successfully detected with good selectivity and sensitivity depending on Δ*R*_CT_ with a detection limit of 1 nM. In addition, the Ap-DNA (1) film was successfully performed in environmental lake water and orange juice samples with good recoveries.

## Materials and methods

### Materials

The thiolated AP-DNA (1) for ACE [41] and control DNA (2) were chemically synthesized by Shanghai Sangon Biological Engineering Technology & Service Co. Ltd (http://www.sangon.com).

5'-HS-(CH_2_)_6_-TTTTTT***TGTAATTTGTCTGCAGCGGTTCTTGATCGCTGACACCATATTATGAAGA***-3' (AP-DNA (1))

5'-HS-(CH_2_)_6_-TTTTTTTATCGTCAGCAGTTAGCCGTATGATGGCAGCAGTTAGCCGT-3' (Control DNA (2))

Tris (tris-(hydroxymethyl)-aminomethane) (Tris), K_3_[Fe(CN)_6_], K_4_[Fe(CN)_6_], EDTA, NaClO_4_, KCl, MgCl_2_, 6-Mercapto-1-hexanol (6-MCH), tris (2-carboxyethyl) phosphine hydrochloride (TCEP), [Ru(NH_3_)_6_]Cl_2_ and [Ru(NH_3_)_6_]Cl_3_ were obtained from Sigma-Aldrich (http://www.sigmaaldrich.com/china-mainland.html) and directly used without further purification. Acetamiprid (ACE), methyl parathion (PAT), chlorpyrifos (CHP), dipterex (DIP) and atrazine (ATR) were purchased from Aladdin Co., Ltd (https://www.aladdin-e.com/). HCl, HClO_4_, NaCl, CaCl_2_ were purchased from Sinopharm Chemical Reagents Beijing Co., Ltd (http://www.crc-bj.com). The gold working electrodes (99.99% polycrystalline, diameter 1 mm) were purchased from Aida Instrument Inc. in Tianjin (China) (http://www.tjaida.cn).

10 μM DNA stock solution was first prepared in 20 mM Tris-HCl (pH = 7.4), and heated at 80°C for 5 min before cooling down to room temperature. After completion of DNA annealing reaction, 1 μM DNA immobilization buffer (I-DNA) containing 1.0 M NaCl, 1mM EDTA, 1 mM TCEP in Tris-HCl (10 mM, pH 8.0) and the binding buffer (B-buffer) containing 100 mM NaCl, 200 mM KCl, 5 mM MgCl_2_, 1 mM EDTA in Tris-HCl (20 mM, pH = 7.4) were subsequently prepared [2, 38]. Different concentrations of ACE solutions were prepard with the B-buffer. Tropicana orange juice samples were bought in the Chaoshifa supermarket lacated at Zhanghua South Road. Lake water was sampled in the Summer Palace lake (S1 Fig in S1 File) and then filtered with the 0.45 μm filter membrane prior to the ACE "B-buffer" preparation. All other solutions were prepared with Milli-Q water (18.2 MΩ cm resistivity) if not specified.

### Fabrication of the DNA sensor

The electrochemically activated gold electrodes and the thiolated AP-DNA (1) modified electrode films were prepared according to previous methods reported before [40, 42]. After that, the AP-DNA (1) modified sensors were washed with 20 mM Tris-HCl (pH = 7.4) buffer and kept at 4°C prior to use. Each AP-DNA (1) modified electrode can be used only one time.

### Electrochemical measurements

CHI 650D electrochemical workstation (Shanghai Chenhua instrument Co. Ltd., China) was employed to perform EIS measurements with three-electrode system in an electrochemical cell containing 2 mM [Fe(CN)_6_]^3-/4-^ Tris-NaClO_4_(20 mM, pH = 7.4) solution. Briefly, the AP-DNA (1) modified films were incubated with the ACE for certain durations of time, then washed with 20 mM Tris-NaClO_4_ (pH = 7.4) buffer for 30 s to remove any unbound analyte. EIS of the AP-DNA (1) films before and after interaction with ACE were all recorded. All measurements were performed in triplicate.

### Circular circular dichroism spectra measurement

Circular dichroism (CD) spectra measurement was carried out with the Jasco J-810 spectropolarimeter. First, preparing 25 μM AP-DNA (1) solutions in Tris-HCl (20 mM, pH = 7.4) buffer and B-buffer, respectively, then adding 25 μM ACE to the above prepared AP-DNA (1) solutions. Finally, measuring the CD spectra of the prepared two systems after 2 h. The spectra of each sample were accumulated and averaged from three scans. Parameters: λ ranged from 200 to 400 nm, intervals 0.5 nm, scan rate 200 nm min^-1^.

## Results and discussion

### ACE sensing principle

**Scheme 1:**
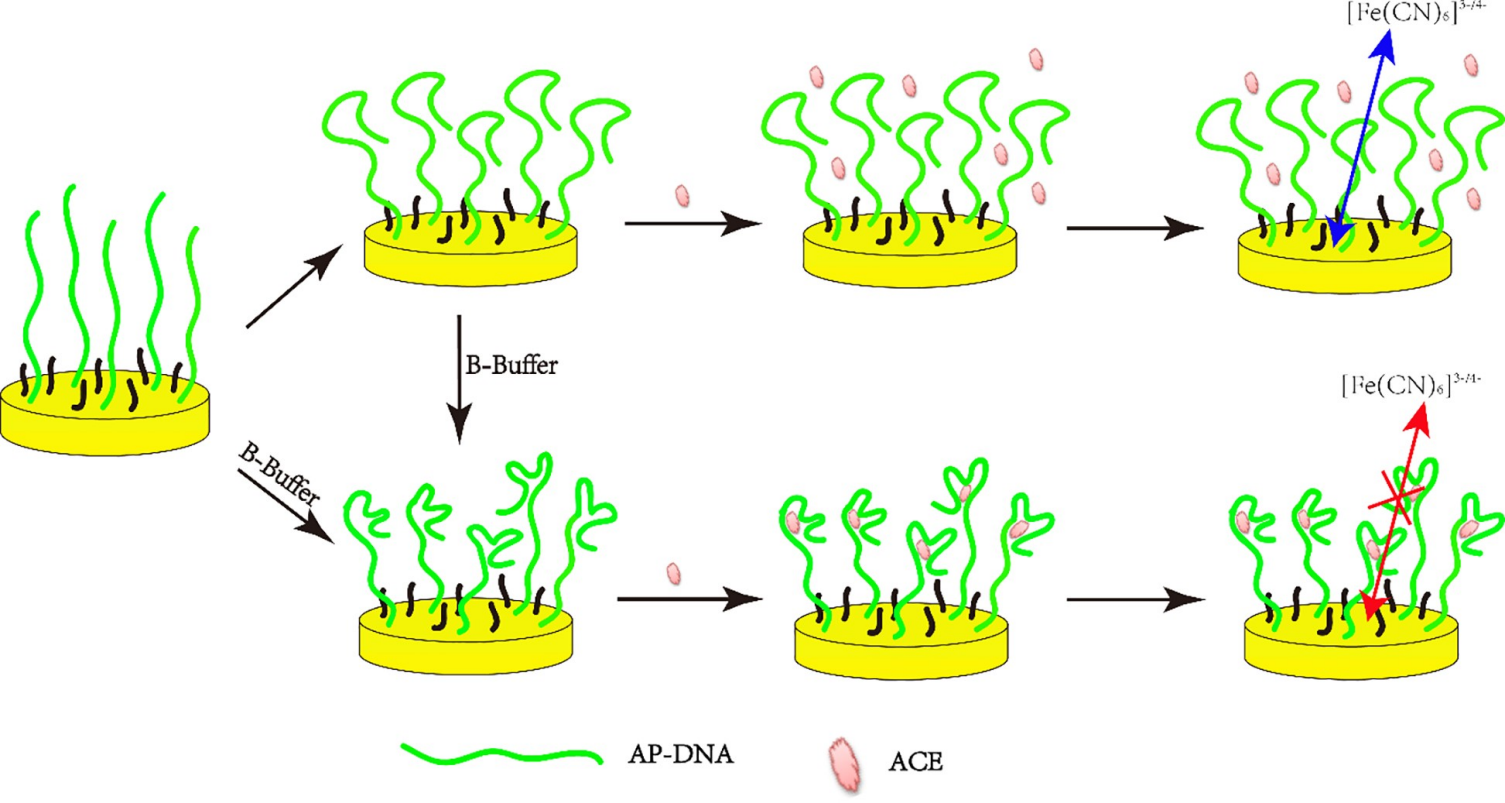
Principle of structure switchable AP-DNA (1) assay for the detection of ACE.

A schematic diagram illustrating the ACE electrochemical sensing principle was shown in Scheme 1. As shown in Scheme 1, the arbitary single strand AP-DNA (1) anchored to the gold electrode surface can form a loosely secondary structure in Tris-HClO_4_ (20 mM, pH = 7.4), which cannot specifically bind with ACE. However, when incubated in B-buffer solution the loosely secondary structure or the arbitary single strand AP-DNA (1) transformed into a more stable hairpin-like structure, forming a stem-loop bulge in the AP-DNA (1), which can serve as the specific recognizing site for ACE [11]. The specific binding interaction between the stem-loop bulge and ACE formed a ACE/AP-DNA (1) complex that hindered the electron transfer and induced the charge-transfer resistance (*R*_CT_) increase between the solution-based redox probe [Fe(CN)_6_]^3−/4−^ and the electrode surface. The change of Δ*R*_CT_ (Δ*R*_CT_ = *R*_CT(after)_-*R*_CT(before)_) is positively related to the ACE level, so the determination of ACE can be quantitatively tested by EIS.

### Demonstration of the sensing principle

**Fig 1:**
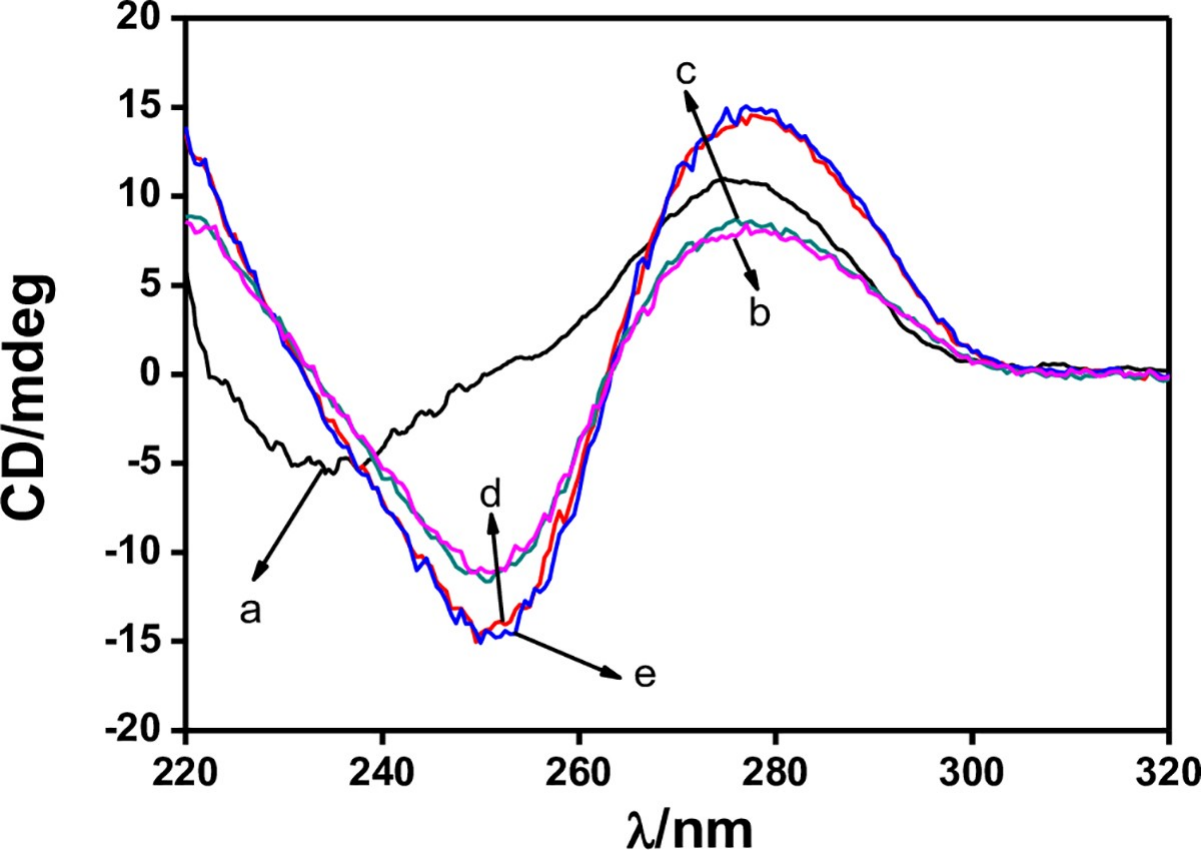
CD measurements: (a) 25 μM DNA (control DNA) in Tris HClO_4_ (20mM, pH 7.4); (b) 25 μM Ap-DNA (1) in Tris-HClO_4_ (20mM, pH 7.4); (c) addition of 25 μM ACE to (b); (d) 25 μM Ap-DNA (1) in B-buffer; (e) addition of 25 μM ACE to (d).

To capture the target molecule, aptamers must change its conformation to a secondary structure with selectively binding site [43], and then binding with the target molecules by forming many weak bonds (such as hydrogen bond interaction and π-stacking interaction) [44]. Studies showed that the CD spectrum can provide a reliable determination of the DNA conformation, and has been widely applied as a very useful tool to study the conformations of DNA in solution. So, CD measurements were first carried out to investigate the proposed sensing mechanism by investigating the conformational switches of the Ap-DNA (1). As shown in Fig 1, the arbitary control DNA (2) showed a negative peak around 235 nm and a positive peak at 272 nm in Tris-HClO_4_ (20 mM, pH = 7.4) (line a), which is the characteristic spectrum of a random coiled single strand DNA as reported before [45]. For AP-DNA (1), the CD spectra showed a positive peak at 277 nm and a negative peak at 250 nm in Tris-HClO_4_ (20 mM, pH = 7.4) (line b), which is the characteristic spectrum of a hairpin-like DNA (containing stem-loop structure) [46]. When instead by B-buffer, a notable increase was observed in the CD intensity at the negative (250 nm) and positive (277 nm) band (line d), proving the random coiled AP-DNA (1) formed a more stable hairpin-like structure. These results indicated that the AP-DNA (1) undergoes a structural change after incubating in Tris-HClO_4_/B-buffer, repectively, forming specific binding site for ACE. However, only minimal CD intensitiy changes at negative 250 nm and positive 277 nm were observed in the ACE/AP-DNA (1) Tris-HClO_4_ system (curve c), demonstrating that binding with ACE has almost no effect on the hairpin structure. Similarly, very small CD change was observed for the ACE/AP-DNA (1) B-buffer system (curve e). Therefore, we can infer that B-buffer can stabilize the hairpin-like structure of the AP-DNA (1), offering the binding site for ACE, but the binding interaction between ACE and AP-DNA (1) can no further induce obviously structural change [47].

**Fig 2:**
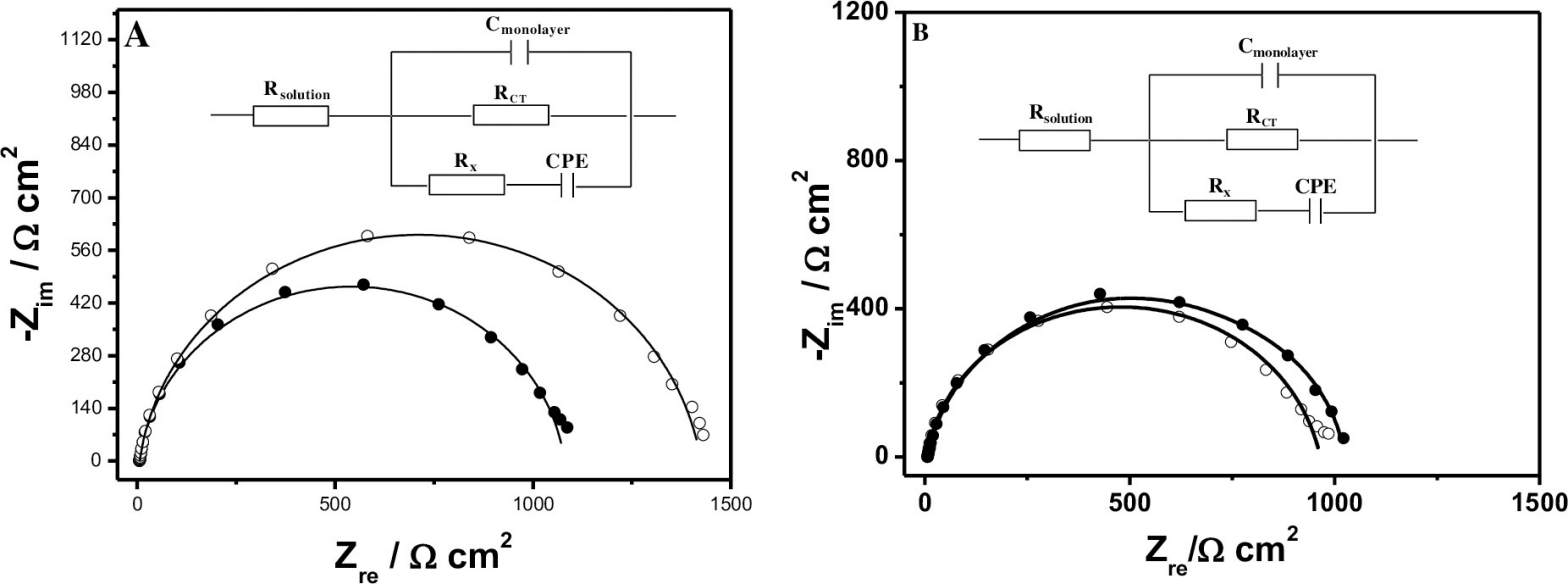
Representative Nyquist plots (−Z_im_ *vs*. Z_re_) for (A) the formed AP-DNA (1) films (white circle) before and after interaction with 50 nM ACE for 40 min (black circle); (B). the formed ss-DNA films (white circle) before and after interaction with 500 nM ACE for 40 min (black circle); Measured data were shown as symbols with calculated fit to the equivalent circuit as solid lines. Inset: The measured data were fit to the equivalent circuit; *R*_solution_, solution resistance; *R*_CT_, charge-transfer resistance; *C*_film_, capacitance of the AP-DNA films; *R*_x_ and CPE, resistance and nonlinear capacitor accounting for 6-mercaptohexanol film.

Next, the specific binding interaction between ACE and AP-DNA (1) was demonstrated by EIS measurements. First, The AP-DNA (1) modified electrode were prepared by immersing the electrodes into the 1 μM AP-DNA (1) immobilizing buffer for overnight (S2 Fig in S1 File). Afterwards, the newly prepared AP-DNA (1) films were reacted with 50 nM ACE in B-buffer containing for 40 min (S3 Fig in S1 File), followed by thoroughly rinsing with Tris-HClO_4_ (20 mM, pH = 7.4) buffer to remove unbound ACE. Before and after incubation in ACE solution, the EIS changes of the AP-DNA (1) films were sequentially recorded in 2 mM [Fe(CN)_6_]^3-/4-^ Tris-NaClO_4_ (20 mM, pH = 7.4) solution to track the ACE binding processes, and the representative Nyquist plots for the corresponding films were shown Fig 2A. The impedance spectra were analyzed by using the modified Randles’ equivalent circuit (inset of Fig 2A).

As shown in Fig 2A, the *R*_CT_ was significantly increased when the prepared Ap-DNA (1) films were incubated in B-buffer (containg 50 nM ACE) for 40 min, confirming the specific binding interaction between the AP-DNA (1) film and ACE. This can be explained that the Ap-DNA (1) with more stable hairpin-like structure on the electrode surface provided the specfic binding sites for ACE, and the formed ACE/AP-DNA (1) complexes then hindered the electron transfer from the solution to the electrode surface [23] (enhanced the repulsion of the redox probe [Fe(CN)_6_]^3-/4-^), resulting in an increased *R*_CT_ [38, 48]. As a control, EIS experiments were also carried out by exploring the DNA (2) modified films reacting with 500 nM ACE in B-buffer (the same procedure as the Ap-DNA (1) film). The representative Nyqiust plots were analyzed and shown in Fig 2B. As shown in Fig 2B, just as expected, there showed only a very minor increase of Δ*R*_CT_ after the control DNA (2) films were utilized over the same time, which can be neglected in camparison with the ACE induced Ap-DNA (1) films Δ*R*_CT_. Therefore, we can infer that the obviously increased Δ*R*_CT_ of the Ap-DNA (1) film was attributed to the specificly capturing ACE by the formed hairpin-like binding sites (stem-loop bulge) on the electrode surface.

Form the above results, we can conclude that: (1) the thiolated single strand Ap-DNA (1) was first modified on the electrochemically activated gold electrodes surface via Au-S bond; (2) the immobilized Ap-DNA (1) folds into a second structure (loosely hairpin-like structure) on the electrode surface in Tris-HClO_4_ (20 mM, pH = 7.4); (3) the loosely hairpin-like structure of the Ap-DNA (1) switched to a more stable hairpin-like structure when incubated in B-buffer; (4) the stem-loop bulge of the hairpin-like Ap-DNA (1), acted as the ACE binding sites, can capture ACE onto the electrode surface and form ACE/AP-DNA (1) complexes, as shown in Scheme 1. Due to the formation of ACE/AP-DNA (1) complexes, *R*_CT_ of the AP-DNA (1) film was greatly increased. Hence, by using Ap-DNA (1) as the specific recognition probe and Δ*R*_CT_ as the output signal, ACE can be electrochemically quantified.

### EIS detection of ACE

Subsequently, the Ap-DNA (1) modified films were utilized for determination of ACE by EIS. The representative Nyquist plots of the specific binding interaction between the Ap-DNA (1) films with 50 nM ACE were measured and shown in Fig 2. The measured impedance spectra were analyzed with the modified Randles’ equivalent circuit (inset of Fig 2). The fitting results were calculated and listed in Table 1.

**Table 1:** Equivalent circuit element fitting results for the AP-DNA (1) films before and after reacting with 50 nM ACE^a^.

	Circuit elements						
	*R*_s_ (Ω·cm^2^)	*C*_film_ (μF·cm^-2^)	*R*_CT_ (Ω·cm^2^)	*R*_x_ (Ω·cm^2^)	CPE (μF·cm^-2^)	*n*	Δ*R*_CT_ (Ω·cm^2^)
**AP-DNA (1) films**	5.5(0.26)	13.9(0.35)	1069.2(16)	3.4(0.31)	11.4(0.36)	0.9(0.1)	0
**+ ACE**	6.1(0.27)	10.0(0.29)	1413.8(13)	2.7(0.25)	10.1(0.41)	0.9(0.1)	344.7(26.2)

^*a*^ The values in parentheses represent the standard deviations from three measurements.

*R*_s_ (solution resistance) is the resistance between the reference electrode and the Ap-DNA (1) films [23], ranging from 5.5 (0.26) Ω·cm^2^ to 6.1 (0.27) Ω·cm^2^. *C*_film_ accounts for the capacitance of the Ap-DNA (1) films on the working electrodes [49], shown in Table 1. It showed that the *C*_film_ decreased after immersing the Ap-DNA (1) films in ACE/B-buffer solution, revealing that the Ap-DNA (1) films binding with ACE might lead to an increase in the film thickness that resulted in a decreased dielectric constant [23]. *R*_x_ and the CPE (constant phase element) accounts for the behavior of the 6-MCH on the working electrode surfaces [50]. CPE accounts for the inhomogeneity of the films on the working electrode surface with the exponential modifier *n* = 0.9 [51]. For ACE quantification, *R*_CT_ is the most important parameter and it represents the charge transfer resistance between the redox probe [Fe(CN)_6_]^3-/4-^ and the gold working electrode surface [52, 53]. As shown in Table 1, after incubating the Ap-DNA (1) films with 50 nM ACE in B-buffer for 40 min, the *R*_CT_ sharply increased from 1069.2 (16) Ω·cm^2^ (*R*_CT(before)_) to 1413.8 (13) Ω·cm^2^ (*R*_CT(after)_) with a Δ*R*_CT_ of 344.7 (26.2) Ω·cm^2^.

**Fig 3:**
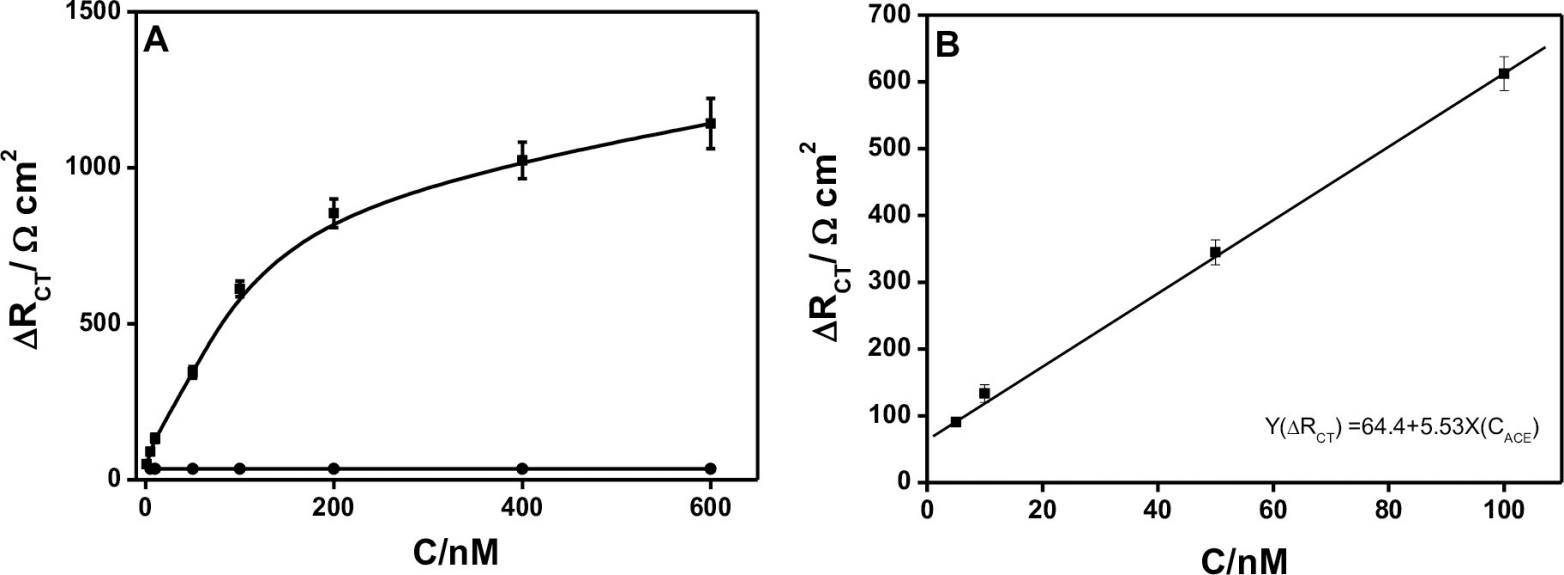
Electrochemical detection of ACE (A): Relationship between Δ*R*_CT_ and the concentrations of ACE (ACE concentrations ranged from 1 nM to 600 nM); (B) Linear relationship between Δ*R*_CT_ and the concentrations of ACE ranged from 5 nM to 200 nM. Error bars are derived from a minimum of three electrodes.

Next, Δ*R*_CT_ was used as the parameter and applied for the ACE detection. First, we exploited the Δ*R*_CT_ changes of the Ap-DNA (1) films after incubation in ACE/B-buffer with increasing the ACE concentrations from 1.0 nM to 600 nM. As shown in Fig 3A, there was a dramatic increase in the Δ*R*_CT_ with increasing the ACE concentrations from 1.0 nM to 200 mM, and then the Δ*R*_CT_ increased slowly from 200 nM to 600 nM. The results indicated that the higher ACE concentration used, the more ACE/AP-DNA (1) complexes formed on the electrode. On the contrary, the Δ*R*_CT_ decreased as decreasing the ACE concentration. No obvious change of Δ*R*_CT_ was observed when decreased to 1.0 nM ACE (compared with the backgrand Δ*R*_CT_ of buffer), and the low detection limit of 1.0 nM was determined. The linear relationship between Δ*R*_CT_ and the concentrations of ACE was in the range of 5.0 nM to 100 nM (shown in Fig 3B), and the fitted regression equation was Y(Δ*R*_CT_) = 64.4+5.53X (*C*_ACE_) (*R*^2^ = 0.9).

Furthermore, the comparison between this method and other approaches for the detection of ACE is listed in Table 2. As shown in Table 2, the proposed assay has distinctive advantages of wide dynamic range, lower detection limit over the reported colorimetric, fluorescent approaches, and it avoids all the complicated modification steps, possessing the merits of simple preparation of the DNA film, less modification steps of the electrode over the nanomaterial modified electrochemical sensors, thus offers a simple, fast and low cost method for ACE detection. Most of all, the results demonstrated that this electrochemical Ap-DNA (1) sensor has great potential to be applied to the AEC detection.

**Table 2:** Comparison of different methods for the detection of ACE.

Sensing methods	Recognition element	Linear range/M	LOD	Ref.
**Colorimetric sensor**	Positively charged AuNPs ((+) AuNPs)	8.7×10^−9^~9.2×10^−7^	0.56 nM	[31]
	Aptamer-wrapped gold nanoparticles	0~5×10^−6^	400 nM	[32]
	Peroxidase-like activity of hemin-rGO composites	1×10^−7^~10×10^−3^	40 nM	[33]
**Fluorescent sensor**	AT-rich dsDNA-templated copper nanoparticles	5×10^−9^~5×10^−7^	2.37 nM	[34]
	Triplex-to-G-qadruplex molecular switch	1×10^−8^~5×10^−7^	2.38 nM	[35]
	Aptamer contained triple-helix molecular switch	1×10^−7^~1.2×10^−6^	9.1 nM	[54]
**Chemiluminescent sensor**	AuNPs-H_2_O_2_-luminol	8×10^−10^~6.3×10^−7^	62 pM	[36]
**Eletrochemical sensor**	3D porous CS/rGO modified electrode	1×10^−11^~1×10^−7^	71.2 fM	[37]
	Au/MWCNT-rGONR modified electrode	5×10^−14^~1×10^−5^	0.017 fM	[38]
	Aptamer/polyaniline and AuNPs modified electrode	2.5×10^−5^~2×10^−5^	86 nM	[39]
	Aptamer/AuNPs modified electrode	5×10^−9^~6×10^−7^	1 nM	[48]
	Aptamer modified electrode	5×10^−9^~2×10^−7^	1 nM	This study

### Performance of selectivity and stability

**Fig 4:**
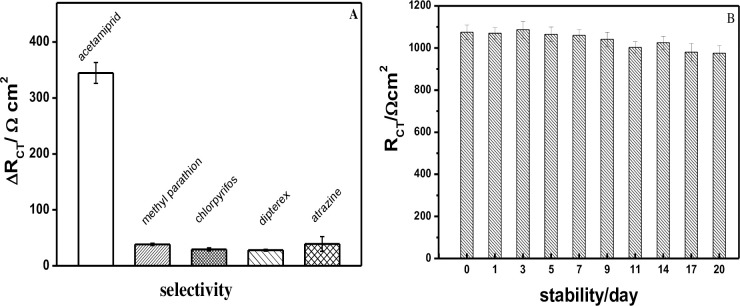
(A) Selectivity of the AP-DNA (1) sensor for ACE (50 nM) and other selected chemicals (such as 50 nM methyl parathion, chlorpyrifos, dipterex, atrazine); (B) Stability of the Ap-DNA (1) film. The Ap-DNA (1) film was incubated in B-buffer and kept at 4°C for 1 to 20 days, and then for EIS measurement. Error bars are derived from a minimum of three electrodes.

Next, we further investigated the selectivity of the Ap-DNA (1) sensor taking Δ*R*_CT_ of the Ap-DNA (1) films as the evaluating indicator. Four pesticides of methyl parathion, chlorpyrifos, dipterex and atrazine were selected, and EIS were carried out to measure *R*_CT_ changes of the Ap-DNA (1) films before and after interacting with the four pesticides. As shown in Fig 4A, ACE caused a considerable increase in Δ*R*_CT_, while the Δ*R*_CT_ changes were all very samll for the selected pesticides (methyl parathion, chlorpyrifos, dipterex and atrazine),demonstrating that the Ap-DNA (1) film showed good selectivity to ACE. As discussed above, the excellent selectivity of this sensor was attributed to the highly specific binding interaction of the stem-loop bulge of Ap-DNA (1) with ACE. Furthermore, compared with the homogeneous assays such as colorimetric, chemiluminescent and fluorescent sensors, the inhomogeneous electrochemical sensor films could be rinsed before and after each step, and suffer less environmental interference [23], which can avoid the case that the signals of colorimetric, chemiluminescent and fluorescent may be quenched and masked by coexisting environment pollutants. The excellent ability of the impedimetric Ap-DNA (1) sensor to distinguish ACE from other pesticides added a novel dimension of selectivity to ACE.

Stability of the immobilized DNA on the electrode surface is one of the outstanding performances for a electrochemical sensor, so EIS of the Ap-DNA (1) films with different storage time (at 4°C) were continually investigated. As shown in Fig 4B, the *R*_CT_ of the newly prepared AP-DNA (1) films was about 1075.3(35) Ω·cm^2^ in 2 mM [Fe(CN)_6_]^3-/4-^ Tris-NaClO_4_ (20 mM, pH = 7.4). As the storage time increases, *R*_CT_ of the AP-DNA (1) films showed a slight trend of fluctuating downward. And only 9.3% of *R*_CT_ was lost for the Ap-DNA (1) films stored for 20 days (957.1 (37.5) Ω·cm^2^). This result indicated that the Ap-DNA (1) film was very stable on the gold electrode due to the strong Au-S covalent bonding. Besides, very small uncertainty values (Error bar) were obtained for EIS measurements, varied from 2.5% to 4.2%, demonstrating good reliability of the sensor films.

### Testing in real samples

In order to validate the feasibility of the assay to detect ACE, the performance of the sensing platform was evaluated with environmental lake water and orange juice samples (purified with 0.45 μM membrane prior to analysis). The recoveries were evaluated by standard addition method, and the experiments were undertaken by spiking the real samples with known concentrations of ACE. As shown in Table 3, excellent spiked recoveries were achieved for ACE detection, with a range of 85.8% to 93.4% for lake water samples and 83.7% to 89.4% for orange juice samples. These results implied that the constructed electrochemical Ap-DNA (1) films were stable, reliable and succesfully applied to real samples analysis, indicating potential application as a good alternative to environmental and food samples for the detection of ACE.

**Table 3:** Analysis of ACE in real samples with different concentrations.

Real Sample	Added (nM)	Detected (nM)	Recovery (%)	RSD (%)
**Lake water**	30.0	25.7	85.8	5.7
	50.0	46.7	93.4	5.2
	80.0	73.0	91.2	3.8
	100.0	92.7	92.7	4.1
**Orange juice**	50.0	41.9	83.7	7.9
	80.0	73.0	87.6	8.3
	100.0	89.4	89.4	6.7

**Fig 5:**
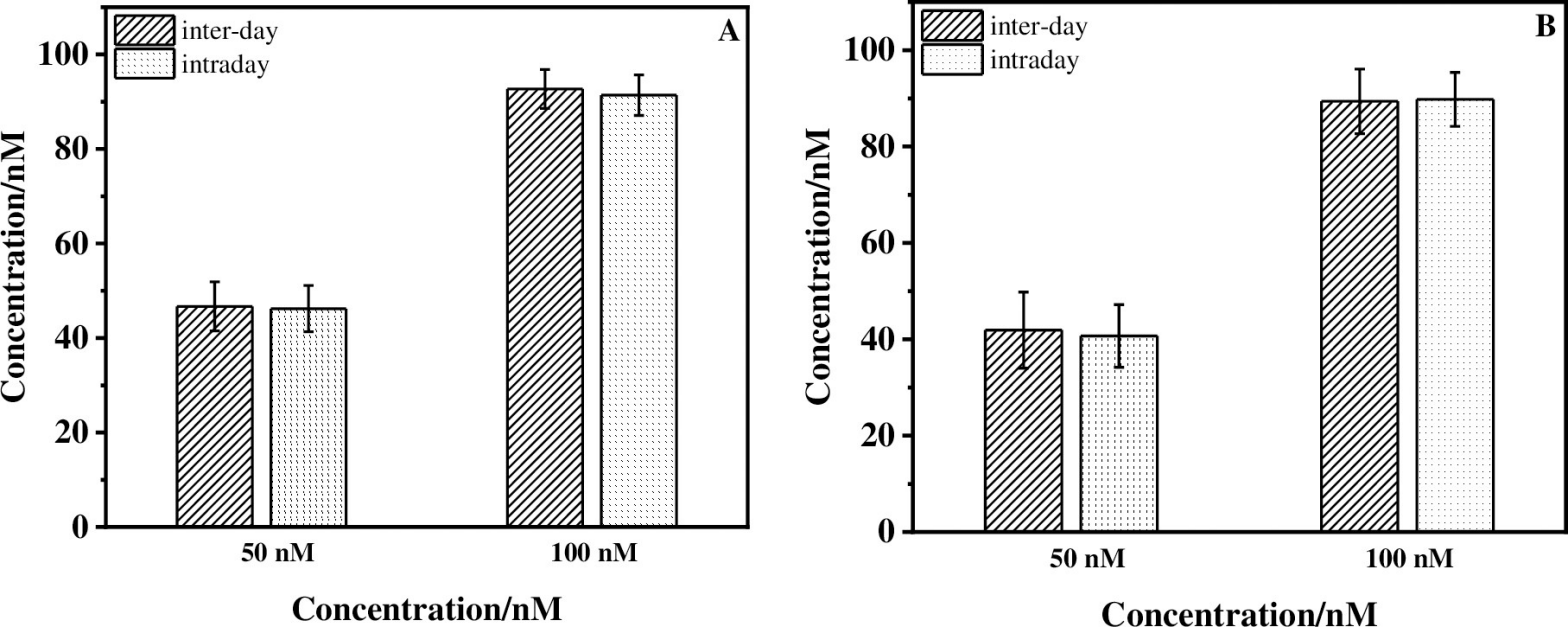
Inter-day/intraday reliability study of the electrochemical AP-DNA (1) sensors for ACE (50 nM/100 nM) detection. (A) Lake water sample; (B) Orange juice sample. EIS measurements for intraday experiment were carried out in the 1, 2, 3, 4 days, respectively. Error bars are derived from a minimum of four electrodes.

Furthermore, an investigation of the inter-day/intraday reliability of the AP-DNA (1) sensor was also performed to further confirm the analytical method. 50 and 100 nM concentrations of ACE in real samples were tested. As shown in Fig 5, there were no obvious differences between the calculated average values obtained from the two different methods (inter-day and intraday EIS measurements) for both the lake water and orange juice samples. These results confirmed the inter-day and intraday reliability of the sensor in real samples.

## Conclusion

In this paper, a label-free impedimetric AP-DNA (1) sensor was reported for the detection of ACE with sensitivity and selectivity. The modified AP-DNA (1) on the electrode surface formed a loosely secondary structure in Tris-HClO_4_, and then switched to a more stable hairpin-like structure with stem-loop bulge in B-buffer, which acted as the ACE binding sites. As a result, with Ap-DNA (1) as a sensing probe, ACE was sensitively detected with a limit of 1 nM. Additionally, the Ap-DNA (1) sensor showed excellent ability to distinguish ACE from other pesticides and practicability to detect ACE in lake water and orange juice, with good recoveries in the range from 83.7% to 93.4%. This sensor is easy, reliable and convenient for the detection of ACE, and enables detection of ACE without any chemical modification or fluorescent labeling of the Ap-DNA (1), demonstrating the AP-DNA (1) sensor possessed great potential applications and promising platform for monitoring ACE in environmental and food samples.

## Supporting information

S1 File (DOCX)
