# Supplementary material for: Label-free hairpin-like aptamer and EIS-based practical, biostable sensor for acetamiprid detection
Source: PLoS One. 2020 Dec 23;15(12):e0244297. doi: 10.1371/journal.pone.0244297 (PMC7757884; doi:10.1371/journal.pone.0244297)
Supplement: S1 File — (DOCX) [file pone.0244297.s001.docx]

**Label-free hairpin-like aptamer and EIS-based practical, biostable sensor for acetamiprid detection**

Jianhui Zhen†^a^, Gang Liang†^b, c, d*^, Ruichun Chen^a^, Wenshen Jia^b, c, d*^

^a^ Shijiazhuang Customs Technology Center P.R. China, Shijiazhuang 050051, Hebei Province, China;

^b^ Beijing Research Center for Agricultural Standards and Testing, Beijing Academy of Agriculture and Forestry Science, Beijing 100097, China;

^c^ Risk Assessment Lab for Agro-products (Beijing), Ministry of Agriculture, Beijing 100097, China;

^d^ Beijing Municipal Key Laboratory of Agriculture Environment Monitoring, PR China;

^*^Corresponding Authors: liangg@brcast.org.cn; Tel.: +86-10-51505523

†These authors contributed equally to this work.

**1 Sampling site map**

**
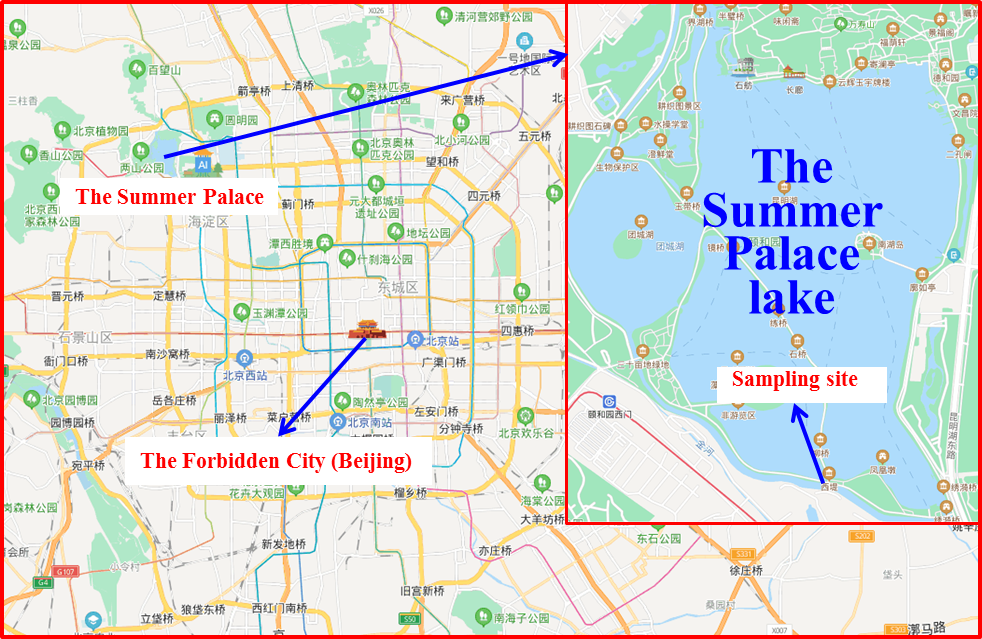
**

**Fig. S1** Location of the sampling site (Obtained from https://map.baidu.com).

The lake water was sampled in the Summer Palace lake, near the scenic spot of West Dyke, and stored in a bottle.

**2** **Immobilization behavior study of AP-DNA (1) on the electrode surface**

In this study, we also investigated the relationship bwtween the AP-DNA (1) density on the electrode surface and the incabation time. The time-dependent response curve of AP-DNA (1) density was shown in Fig. S2. The probe AP-DNA (1) density at the electrode surface was determined by chronocoulometric (CC) method previously reported by Keighley [1]:

*ΔQ=*nFA*Г*_0_ (1-1)

*Г*_DNA_=*Г*_0_ (z/m) (1-2)

*ΔQ*: the intercept difference of chronocoulometric response curves for the AP-DNA (1) film modified electrode in the absence and presence of Ru(NH_3_)_6_^3+^;

*Γ*_AP-DNA_: the probe AP-DNA (1) surface density (mol cm^2-^);

m: the number of phosphate groups on the probe AP-DNA (1);

z: the charge of the redox probe molecule (Ru(NH_3_)_6_^3+^);

n: the number of electrons transferred;

F: the Faraday constant;

A: the electrode area (cm^2^).

**Fig. S2** Relationship bwtween the AP-DNA (1) density and the incabation time. (Condition: 100 μM Ru(NH_3_)_6_^3+^; 20 mM Tirs-HClO_4_ (pH 7.4) buffer). Error bars were derived from a minimum of three electrodes.

As shown in Fig. S2, the AP-DNA (1) density on the gold electrode surface was significantly increased within 3 h when the electrochemically cleaned gold electrodes were incubated in the AP-DNA (1) (1 μM) immobilization buffer. Afterwards, no obvious density change was observed until 15 h. However, to get better modified film, an overnight incubation time was chosen for AP-DNA (1) modification in the present study.

**3 Optimization of the incubation time**

To get better detection results (detection limit), the ∆*R*_CT_ change as a function of incubation time at the AP-DNA (1) films modified electrode was explored. As shown in Fig. S3, the Δ*R*_CT_ of the AP-DNA (1) film was significantly increased after incubated in 100 nM ACE in B-buffer within 40 min until the inflection point appeared. After that, the Δ*R*_CT_ changed slowly with the increased time. Therefore, 40 min interaction time was used in the present study. In addition, the increased ∆*R*_CT_ also confirmed the specific binding interaction of ACE with the AP-DNA (1) on the films.

**Fig. S3** Optimization of the specific binding time of ACE with AP-DNA (1) film. The time-dependent Δ*R*_CT_ changes at the hairpin-like AP-DNA (1) film in the presence of 100 nM of ACE in B-buffer. Error bars were derived from three electrodes.

**References**

[1] S.D. Keighley, P. Li, P. Estrela, P. Migliorato. *Biosens. Bioelectron.*, 2008, 23, 1291-1297.
